# The effect of ostracism on social withdrawal behavior: the mediating role of self-esteem and the moderating role of rejection sensitivity

**DOI:** 10.3389/fpsyg.2024.1411697

**Published:** 2024-08-06

**Authors:** Yuju Lei, Mingyue Li, Chen Lin, Chenshiyuan Zhang, Zhen Yu

**Affiliations:** School of Education, Hubei University of Arts and Science, Xiangyang, China

**Keywords:** ostracism, social withdrawal, self-verification, self-esteem, rejection sensitivity

## Abstract

Extant studies have empirically tested the main two behavior responses following ostracism: prosocial or antisocial. Few studies have investigated the relationship between ostracism and social withdrawal. According to the temporal need-threat model and the self-verification theory, the present study aimed to examine the influence mechanism of ostracism on social withdrawal, especially the mediating role of self-esteem and the moderating role of rejection sensitivity. A total of 1,315 Chinese high school students (52.6% female) completed a written questionnaire. Results showed that ostracism was positively correlated with social withdrawal. Ostracism not only directly predicted social withdrawal, but also indirectly affected social withdrawal by threatening adolescents’ self-esteem. High rejection sensitivity may help aggravate adolescents’ self-esteem threaten perceive from ostracism. Adolescents with high rejection sensitivity felt a greater threat to self-esteem when ostracized. Findings suggest a new direction for understanding individuals’ responses to ostracism.

## Introduction

Ostracism is a ubiquitous negative interpersonal experience when an individual is rejected, ignored or excluded by a group or others (Williams, 2009; Nezlek et al., 2012; Riva and Eck, 2016). Ostracism is a distressing and painful experience that can lead to psychological adjustment problems, such as depression and low self-esteem (Williams, 2009; Platt et al., 2013; Niu et al., 2016), increased health-relevant inflammatory response (Eisenberger et al., 2003; Slavich et al., 2010; Eisenberger and Cole, 2012) and aggressive behaviors (Twenge et al., 2001; Leary et al., 2006; Ren et al., 2018). These problematic outcomes necessitate research into how individuals respond to ostracism. According to the temporal need-threat model of ostracism (Williams, 2009), ostracized individuals may choose pro-social behavior or antisocial behavior, or withdrawal from social interactions as the responses to ostracism (Ren et al., 2016; Ren and Evans, 2020). Compared with the prosocial and antisocial behavioral, withdrawal as an additional behavioral response to ostracism has received few attention (Wesselmann et al., 2015). It has been proposed that if individuals do not have good peer social relationships, they may choose to withdraw from interpersonal situations and use solitude as a possible means of self-protection when they have been ostracized (Chen et al., 2023). Previous studies have found that if individuals continue to associate with people who have rejected them, they will increase the risk of being rejected again in the future (Ladd et al., 2014; Wesselmann et al., 2015). That is, ostracism will induce individuals to stay away from others who have rejected them, even others who have not harmed them. Unfortunately, the withdrawal reactions will induce further ostracism (Ren et al., 2020; Ren and Evans, 2020), and this vicious circle will result in individuals’ interpersonal dysfunction. Ostracized adolescents withdraw from peer interactions, avoiding the situation of ostracism, but also reducing the opportunities for positive interaction with others. The lack of positive interpersonal interactions, can lead to more social skills deficits, induce further ostracism, and increase the risk of depression (Spinhoven et al., 2011). In addition, when adolescents adopt social withdrawal coping styles, isolation from the peer environment increases loneliness, which in turn increases the risk of depression (Katz et al., 2011). Therefore, it is of great importance to reduce the possibility of social withdrawal among adolescents and to clarify the mechanism of social withdrawal among individuals after experiencing ostracism.

### Behavioral responses following ostracism

According to the temporal need-threat model (Williams, 2009), there are three stages of reactions to ostracism: reflexive, reflective, and resignation stage. In the reflexive stage, ostracism threatens basic needs such as belonging, self-esteem, control and meaningful existence. And the immediate responses to ostracism are negative affect and lowered self-esteem (Richman and Leary, 2009). In the reflective stage, ostracized individuals usually cope with three behavioral patterns: pro-social, antisocial, and socially withdrawal (Ren et al., 2016, 2018). How people respond is related to the expectation of the social bond being repaired, the value of the relationship, and the perceived unfairness of ostracism as well as the chronicity and the cost of the ostracism (Richman and Leary, 2009; Williams and Nida, 2011). Individuals may seek social affiliation and become more pro-social in order to increase the likelihood of reconnection (Maner et al., 2007). If the ostracism experience seems unfair or unjust, antisocial responses are likely to follow. Finally, if the ostracism appears chronic, individuals will suffer the third stage of resignation. The chronic ostracism may motivate individuals to withdraw socially, feel alienated and helpless to avoid future ostracism (Williams, 2009; Wesselmann et al., 2015).

### Ostracism and social withdrawal

In addition, it’s worth noting that, although a person may be exposed to ostracism across all ages, adolescents have been found to be more sensitive to rejection and ignorance than adults in daily life (Higgins et al., 2010; Pharo et al., 2011). Compared with adults, adolescents are more likely to choose maladaptive behaviors (hostile aggression or social withdrawal) to cope with ostracism (Bowker et al., 2011). China is a collectivist country. In this cultural context, adolescents are deeply dependent on peer relationships. Therefore, adverse peer relationship experiences (rejection, ostracism) may be the cause and consequence of adolescents’ social withdrawal (Bowker et al., 2011; Ladd et al., 2014). There is a positive correlation between ostracism and social withdrawal in adolescents (Bowker and Raja, 2011; Chen et al., 2023). Empirical studies have proved that social withdrawal is the third behavioral reaction of ostracism after prosocial and antisocial (Ren et al., 2016).

Why do ostracized adolescents choose to withdraw from social situation? Studies have also shown that adolescents’ responses to ostracism were related to the attribution of ostracizer, and the possibility of re-affiliating with the ostracizer or other potential partner (Maner et al., 2007; Richman and Leary, 2009; Williams and Nida, 2011). In the reflective stage, if individuals make hostile attributions to ostracizers, or negative cognitive believes about themselves (e. g., clumsy, unpopular or incompetent), they are more likely to withdraw from peer interaction (Ladd et al., 2014). In addition, according to the Social-cognitive Theory (Crick and Dodge, 1994), ostracism triggers individuals’ hostile expectations in social activities (Crick and Ladd, 1993; DeWall et al., 2009), which makes them difficult to establish intimacy relationships with others, so they attempt to avoid relationships (Ren et al., 2018). In the resignation stage, individuals tend to withdraw from interpersonal relationship due to chronic and extreme pain and pressure in a negative disposition (Williams, 2007). The withdrawal response is thought to be caused by a combination of cognitive (e.g., “I am unpopular”), emotional (e.g., shame), and physiological changes (e.g., inflammation) (Slavich et al., 2010). Ostracized individuals may choose to withdraw from interpersonal situations as a possible means of self-protection to ease the pain of ostracism (Richman and Leary, 2009; Wesselmann et al., 2014). Therefore, hypothesis 1 of this study was established: Ostracism could significantly predict adolescents’ social withdrawal behaviors.

### Self-esteem as a potential mediator

Self-esteem is a person’s general sense of worth (Rosenberg, 1965), and it is a psychological cognitive mechanism for an individual to adapt to social culture (Baumeister and Leary, 1995). Self-esteem is an important component of self-concept, and its formation, development and change are significantly related to past experiences of individuals (Leary et al., 1995). According to the self-verification theory, individuals continuously accept and integrate external information in the process of self-concept formation (Korman, 1970; Swann, 1983, 1997). Self-verification theory holds that individuals tend to maintain their existing self-concepts and they will act in accordance with their self-concepts (Swann, 2012). In the process of self-verification, individuals with negative self-concept expect others to view them negatively (Swann et al., 2007). In the context of ostracism, individuals who are ostracized receive negative feedback from others, which makes them more convinced that they are unwelcome, that is, they realize self-verification. In accordance with self-verification model, as an important of self-concept, self-esteem is a potential variable to influence individuals’ behavioral development. When individuals are ostracized, their self-esteem is threatened and they will doubt their sense of worth (Li et al., 2019). Ostracized individuals believe that they lack communication skills and are less valuable (Ferris et al., 2015; Kong, 2016), and tend to withdraw from interpersonal situations. Actually, self-esteem has been widely documented as a mediating role accounting for the effects of ostracism on behavioral responses (Chung and Yang, 2017; Li et al., 2019). Based on the literature reviewed above, this study proposed the second hypothesis: The self-esteem would play a “bridge” role between ostracism and social withdrawal, that is, self-esteem mediated the effect of ostracism on social withdrawal.

### The moderate role of rejection sensitivity

Why does ostracism cause trouble for some people while leaving others unscathed? What are the moderating factors? Through literature review, it was found that “rejection sensitivity” was an effective predictor of withdrawal behavioral response to ostracism (Zimmer-Gembeck, 2015; Zimmer-Gembeck et al., 2021). Rejection sensitivity is defined as a heightened anxious expectation of rejection in social situations (Downey and Feldman, 1996; Ding et al., 2020). Within the Rejection Sensitivity Model, the rejection sensitivity results from individuals’ negative sociocultural experiences (rejection, ostracism, and victimization) (Downey and Feldman, 1996; Zimmer-Gembeck, 2016; Gao et al., 2021). And the rejection sensitivity model emphasizes how rejection sensitivity affects the individuals’ responses to interpersonal interactions (Zimmer-Gembeck and Nesdale, 2013; Gao et al., 2017; Zimmer-Gembeck et al., 2022). When in a negative social situation, individuals high in rejection sensitivity are more likely to adopt adverse cognitive reactions (e. g., self-blame), affective reactions (e.g., anxiety, sadness, and anger) and maladaptive behavioral responses (e.g., withdrawal and aggression) (Watson and Nesdale, 2012; Zimmer-Gembeck et al., 2022). Additionally, previous research has shown that rejection sensitivity disrupts the social repair processes following a painful ostracism (Barton et al., 2023). It is reasonable to predict that individuals with high rejection sensitivity are more likely to adopt social withdrawal coping with ostracism. Therefore, hypothesis 3 was proposed that the rejection sensitivity would moderate the relationship between ostracism and social withdrawal.

In addition, according to the Trait Activation Theory (Tett and Burnett, 2003) and the Cognitive-Affective Processing System Framework (Mischel and Shoda, 1995), when encountering negative social situations, individuals’ threat expectations are easily activated. High rejection sensitivity may help aggravate adolescents’ self-esteem threaten perceive from ostracism. Individuals with high rejective sensitivity may perceive lower self-assessment and threaten their self-esteem due to their negative social relationship expectations (Li et al., 2019). Some studies have found that individuals with high rejection sensitivity ostracized by peers, they may fall into rumination (Pearson et al., 2011), which causes more damage to self-esteem, and further increases social anxiety and social withdrawal behaviors. Based on the self-validation theory (Swann, 1983, 2012), the rejection sensitivity may induce the negative self-verification process of individuals too. If low self-esteem individuals also have high rejection sensitivity traits, they will be more sensitive to negative social evaluation, leading to more social withdrawal behaviors. Based on the above analysis, this study proposed hypothesis 4 and hypothesis 5.

*Hypothesis 4*: The rejection sensitivity would moderate the relationship between ostracism and adolescents’ self-esteem.

*Hypothesis 5*: The rejection sensitivity would moderate the relationship between self-esteem and social withdrawal.

## The current study

**Figure 1:**
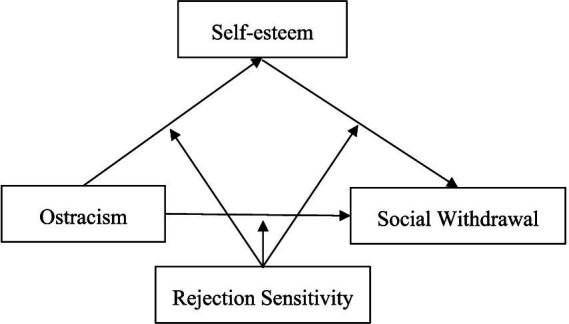
The hypothesized moderated mediation model.

China is a collectivist country. In this cultural context, Chinese adolescents have introverted personality traits, which may promote them to choose social withdrawal and enhance interpersonal difficulties (Chen et al., 2023). Taken together, ostracism is associated with social withdrawal, however, studies on this topic are rather limited. In fact, exploring the relationship between ostracism and social withdrawal behavior will help us to deepen our understanding of the behavioral response to ostracism. Thus, the present study tested the relationship between ostracism and social withdrawal, and the mediating role of self-esteem and the moderating role of rejection sensitivity in this model (see Figure 1).

## Methods

### Participants and procedure

All procedures were reviewed and approved by the authors’ University Ethical Committee. The study was conducted with the informed consent of teachers and principals. In addition, the participants were high school students, whose average age was 16.19, with certain cognitive and behavioral abilities. Therefore, the study was conducted with informed consent of all participants. A total of 1,380 students voluntarily participated in the present study. The convenient sampling method was used to select students from three public high schools in Xiangyang and Qingdao in China. The economic and cultural development of these cities was at a moderate level. After excluding invalid data (answering regularly), the final sample included 1,315 students (52.93% female). The students ranged in age from 15 to 18, with an average age of 16.19 (*SD* = 0.94). As for the sample, 545 students were freshmen, 452 students were sophomores, and 318 students were seniors.

The study relies on self-reporting, and participants may choose more ethical options due to the social approval effect. To solve this problem, questionnaires were set to be anonymous, some items were controlled by reverse scoring, and the researchers promised to keep any information about the participants confidential. The data were collected in the form of paper-and-pencil in classrooms, during the noon break which is about an hour. The participants had plenty of time to complete all questionnaires, and most of participants took about 15 min to complete all questionnaires. All participants were free to withdraw from the study at any time.

### Measures

#### Perceived ostracism

The Chinese version of Ostracism Experience scale for Adolescents (OES-A) (Gilman et al., 2013; Zhang et al., 2018) was administered to measure adolescents’ perceptions of being ignored or excluded by others. This scale contains a total of 11 items, two dimensions of being ignored and excluded (e.g., perceptions of being ignored “in general, others treat me as if I am invisible”; perception of being excluded “In general, others invite me to join their club, organization, or association”). Participants rated the items using a 5-point Likert scale from 1 = “never” to 5 = “always.” Higher scores indicate higher levels of perceived ostracized experience. The scale has demonstrated good reliability and validity among Chinese adolescents (Zhang et al., 2018). The results of confirmatory factor analysis showed that the fit indices of the scale were excellent (*χ^2^/df* = 2.11, CFI = 0.98, TLI = 0.98, RMSEA = 0.04). Cronbach’s *α* of the questionnaire in the present study was 0.89.

### Self-esteem

The Rosenberg Self-esteem Scale developed by Rosenberg (1965) was used to test adolescents’ level of self-esteem. It contains a total of 10 items, such as “I take a positive attitude toward myself.” This questionnaire is the most widely used tool in the study of self-esteem, and has shown good reliability and validity in Chinese culture (Li et al., 2019). The scale uses 4-point Likert scale (1 = very inconsistent, 4 = very consistent). Higher scores indicate higher the level of self-esteem. In this study, the Cronbach’s *α* was 0.88.

### Rejection sensitivity

The Chinese version of the Tendency to Except Rejection Scale (TERS) (Jobe, 2003) was administered to test the level of anxiety and the expectation to be rejected. The scale includes 18 items and the scoring method is 5-point Likert scale (1 = very inconsistent, 5 = very consistent). The previous studies have shown that TERS has good reliability and validity in the Chinese context (Li, 2007). In this study, the Cronbach’s α coefficient of the scale was 0.84.

### Social withdrawal

The Chinese version of the Social Avoidance and Distress Scale (SAD) (Watson and Friend, 1969) was administered to test adolescents’ intention of social withdrawal. The scale includes two dimensions of social withdrawal and social anxiety, and consists of 28 items. The scale uses 5-point Likert scale (1 = very inconsistent, 4 = very consistent). In this study, Cronbach’s *α* coefficient of this scale was 0.89.

### Data analytic procedures

In this study, all the data analyses were conducted with the SPSS 20.0 software package. Pearson correlation analyses with bias-corrected and accelerated bootstrap with 5,000 bootstrap samples were used to explore the relationships among research variables. The hypothesized moderated mediation model was tested in SPSS using the PROCESS macro (Model 8) developed by Hayes (2013). All the variables in regression models were standardized.

## Results

### Common method deviation and covariance test

To avoid common method biases caused by self-reporting, appropriate procedural and statistical controls were made in this study. In the procedure, questionnaires were set to be anonymous, some items were reversed scored. In terms of statistics, Harman single factor method, a confirmatory factor analysis and controlling unmeasured single method latent factor method were used to analyze and test the common method bias of the data (Podsakoff et al., 2003; Zhou and Long, 2004). First, the results of Harman’s one-way test showed that there were nine factors with feature roots greater than 1, and the cumulative variance explained by the first factor was 26.45%. It was less than the critical value of 40%, indicating that there was no serious common method bias in the data of this study. Second, we conducted a confirmatory factor analysis to establish discriminant validity of the measures. When all items were loaded onto their corresponding latent constructs, the CFA model failed to converge, likely due to a large number of items in the measurement of rejection sensitivity and social withdrawal. Thus, we created some parcels for large number of items, such as creating six parcels for 18 items of rejection sensitivity. The results revealed that the one-factor model fit (*χ^2^/df* = 22.04, CFI = 0.58, TLI = 0.56, RMSEA = 0.15) was significantly worse than the fit of the 4-factor model used in this study (*χ^2^/df* = 2.90, CFI = 0.94, TLI = 0.95, RMSEA = 0.06). The results demonstrated that this study had no serious common method bias. Third, this study adopted the “control unmeasured single method latent factor method,” in which all the items were loaded onto their corresponding latent variables. At the same time, these items were also loaded onto a common variable to compare whether the model fit after controlling for the common method factor was better than the original model. Comparing the fit indices of two models: 1*χ^2^/df* = 0.09, 1CFI = 0.01 and 1TLI = 0.01, 1RMSEA = 0.005, the difference between two models was not significant. The difference was <0.02 for both CFI and TLI, and <0.01 for RMSEA. The model controlling for common method bias was not significantly better than the original model. In summary, these results demonstrated that there was no serious common method bias in the data of this study.

### Descriptive statistics and correlation

Analysis of descriptive statistics, including correlation, mean, and standard deviation among the variables, were shown in Table 1. Ostracism was positively correlated with rejection sensitivity and social withdrawal (*r* = 0.36, *r* = 0.63, *p* < 0.01). Ostracism was negatively correlated with self-esteem (*r* = −0.59, *p* < 0.01). Rejection sensitivity was negatively correlated with self-esteem (*r* = −0.43, *p* < 0.01), and rejection sensitivity was positively correlated with social withdrawal (*r* = 0.28, *p* < 0.01). Self-esteem was negatively correlated with social withdrawal (*r* = −0.55, *p* < 0.001).

**Table 1:** Correlation, means, and standard deviations.

	*M*	*SD*	1	2	3	4	5	6
1. Gender	1.53	0.49	1.00					
2. Age	16.19	0.94	0.02	1.00				
3. Ostracism	2.47	0.73	−0.10^*^	0.14^*^	1.00			
4. RS	2.97	0.83	0.06	0.09	0.36^**^	1.00		
5. Self-esteem	3.31	0.59	0.01	−0.17^*^	−0.59^**^	−0.43^**^	1.00	
6. SW	2.94	0.55	0.03	0.18^*^	0.63^***^	0.28^**^	−0.55^***^	1.00

*N* = 1,315. Gender (male = 1; female = 2). ^***^p < 0.001, ^**^p < 0.01, ^*^p < 0.05; M, mean; SD, standard deviation; RS, rejection sensitivity; SW, social withdrawal.

### Testing for hypothesized moderated mediation model

Two regression models (see Tables 2, 3) were used to analyze the mediating effect of self-esteem between ostracism and social withdrawal (Model 4) and the moderating effect of rejection sensitivity (Model 59).

**Table 2:** Regressions testing the mediation model.

Dependent	Independent	*R*	*R^2^*	*F*	*β*	*t*
Self-esteem	Ostracism	0.60	0.36	245.91	−0.59	−26.13^***^
	Gender				−0.05	−2.34^*^
	Age				−0.08	−3.73^*^
SW	Ostracism	0.68	0.46	273.87	0.48	18.62^***^
	Self-esteem				−0.26	−10.04^***^
	Gender				0.08	4.07^**^
	Age				0.06	2.97^*^

^***^p < 0.001, ^**^p < 0.01, ^*^p < 0.05; SW, social withdrawal.

**Table 3:** Regressions testing the moderated mediation model.

Dependent	Independent	*R*	*R^2^*	*F*	*β*	*t*
Self-esteem	Ostracism	0.65	0.42	189.06^***^	−0.48	−20.61^***^
	RS				−0.25	−10.94
	Ostracism ×RS				−0.09	−4.71^*^
	Gender				−0.04	−1.76
	Age				−0.07	−3.50
SW	Ostracism	0.68	0.46	158.98^***^	0.48	18.61^***^
	Self-esteem				−0.26	−9.86^***^
	RS				−0.02	−0.74
	Ostracism × RS				−0.01	−0.59
	Self-esteem × RS				0.0002	0.01
	Gender				0.07	3.28
	Age				0.08	4.03^*^

RS, rejection sensitivity; SW, social withdrawal. ^*^p < 0.05, ^***^p < 0.001.

The test results of the mediating effect of self-esteem are shown in Table 2 (Model 4). After controlling gender and age, ostracism directly predicted the social withdrawal (*β* = 0.63, 95% CI = [0.59, 0.67], *p* < 0.001). When self-esteem was included, the direct effect of ostracism was still significant (*β* = 0.48, 95% CI [0.43, 0.53], *p* < 0.001), and ostracism significantly negatively predicted self-esteem (*β* = −0.59, 95% CI [−0.63, −0.54], *p* < 0.001). Self-esteem significantly negatively predicted social withdrawal (*β* = −0.26, 95% CI [−0.31, −0.21], *p* < 0.001). In addition, the mediating effect value of self-esteem was 0.15, and its Bootstrap 95% CI [0.12, 0.19] did not include 0, indicating that self-esteem played a partial mediating role between ostracism and social withdrawal.

### The moderating effect of rejection sensitivity

**Figure 2:**
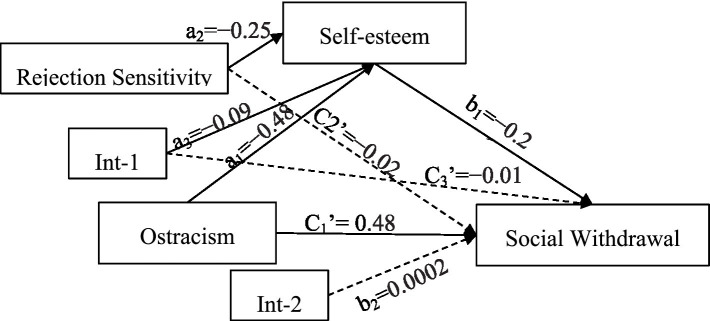
Statistical diagram of the conditional process model. *n* = 1,315. Int_1, ostracism × rejection sensitivity; Int_2, self-esteem × rejection sensitivity.

In addition, the test results of the moderated mediation model are shown in Table 3 and Figure 2. When rejection sensitivity was included in the model, the interaction term of ostracism and rejection sensitivity had a significant predictive effect on self-esteem (*β* = −0.09, 95% CI = [−0.13, −0.05], *p* < 0.001). This result supported hypothesis 4, that rejection sensitivity played a moderating role between ostracism and self-esteem, namely, the first half of mediating effect. However, the hypothesis 3 was not supported, the interaction term of ostracism and rejection sensitivity had no significant predictive effect on social withdrawal.

**Figure 3:**
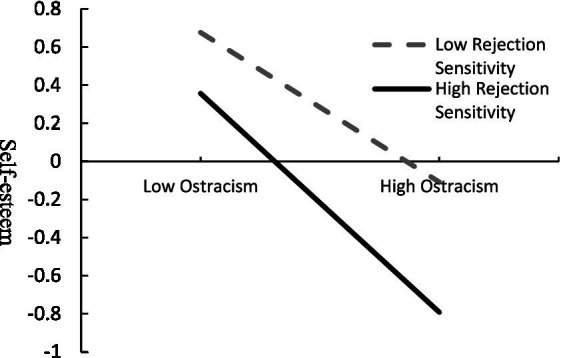
Simple slope diagram for moderating effects.

These interactions were further examined using simple slope analysis. We tested the predicted effect of the ostracism on self-esteem among three different values of rejection sensitivity (i.e., *M* – 1 *SD* and *M* + 1*SD*). Results indicated that, compared with low rejection sensitivity (*M* − 1*SD*), high rejection sensitivity (*M* + 1*SD*) had a greater mediating effect of self-esteem on social ostracism and social withdrawal (effect value was 0.15). Although ostracism could predict the self-esteem when the rejection sensitivity was −1(*β* = −0.39, *t* = −12.12, *p* < 0.01); 95% CI = [−0.45, −0.33], the effect value was smaller than when the rejection sensitivity was 1 (*β* = −0.57, *t* = −20.78, *p* < 0.01); 95% CI = [−0.63, −0.52]. Results indicated that high rejection sensitivity will aggravate the negative effects of ostracism on self-esteem (see Figure 3 for the specific moderating effect). In situations of low ostracism, individuals with high rejection sensitivity also experience more self-esteem threats, interpret ambiguous social situations as hostile, unfriendly than individuals with low rejection sensitivity. When confronting chronic ostracism, compared with individuals with low rejection sensitivity, individuals with high rejection sensitivity have lower self-evaluation, and social rejection has a greater threat to their self-esteem.

## Discussion

This study focused on why and how individuals choose social withdrawal response to ostracism, and investigated the mediating and moderating effects of self-esteem and rejection sensitivity. The results showed that adolescents tended to withdraw from social situation when confronting ostracism. Self-esteem partially mediated the relationship between ostracism and social withdrawal. The first half of the mediating effect of self-esteem was moderated by the rejection sensitivity. These findings broaden existing research on the relationship between ostracism and behavioral response. Besides, this study revealed the self-esteem and the rejection sensitivity affect the relationship between ostracism and social withdrawal. It can further provide a basis for promoting the active intervention of ostracism and social withdrawal.

### Theoretical implications

Extant studies have empirically tested the main two behavior responses following ostracism: prosocial or antisocial. The present study investigated the relation between ostracism and social withdrawal based on the temporal need-threat model and the self-verification theory. And the findings broaden existing research on the relationship between ostracism and behavioral response. Results indicated that adolescents who perceived ostracism were more likely to be socially disadvantaged and withdraw from social situations, which supported the hypothesis 1. This is consistent with previous findings that Chinese adolescents might choose withdrawal behavior due to the introverted interpersonal traits (Chen, 2010; Chen et al., 2023). The present study found that self-esteem partially mediated the relationship between ostracism and social withdrawal, which generally supported the core hypothesis of the temporal need-threat model of ostracism and self-verification theory. That is, ostracism is an important risk factor for self-esteem (Williams, 2009). The feeling of being ignored and rejected by ostracism conveys a negative evaluation to adolescents, which they internalize as part of their self-concept, leading to the reduction of their sense of self-worth and meaning (Korman, 1970; Swann, 1983, 1997; Baumeister and Leary, 1995). Self-esteem reflects a generalized sense of eligibility for relationships, and self-esteem is a potential variable to influence behavioral development. Self-verification theory holds that individuals tend to maintain their existing self-concepts and they will act in accordance with their self-concepts (Korman, 1970; Swann, 2012). As an important element of self-concept, self-esteem is a potential variable to influence individuals’ emotional and behavioral responses. When individuals are ostracized, their self-esteem is threatened and they will doubt their sense of worth (Ferris et al., 2015; Kong, 2016). Ostracized individuals believe that they lack communication skills and are less valuable, and tend to withdraw from social activities. Individuals choose social withdrawal to cope with ostracism, which will verify their self-cognition of low self-esteem, and further increase their risk of being ostracized. That is, withdrawal behavioral reactions in adolescents are likely to enhance experiences of being ostracized resulting in a vicious circle of ostracism (be ostracized- self-esteem threat- social withdrawal-be ostracized) (Levy et al., 2001). And these behavioral reactions can lead to dysfunctional relationship of adolescents (Meehan et al., 2018). The results of this study extend the applicability of the temporal need-threat model of ostracism and the self-validation theory. They also provide support to clarify the influencing process of ostracism on social withdrawal in adolescents, and provide clues for the development of intervention strategies to reduce the negative effects of ostracism.

The results of this study also indicated that the relationship between ostracism and self-esteem was moderated by the rejection sensitivity. When ostracized, adolescents with high rejection sensitivity felt more self-esteem threats than those with low rejection sensitivity. In low ostracism situations, even in ambiguous interpersonal situations, individuals’ threat expectations and physiological were easily activated due to high rejection sensitivity. They tend to believe that they will be rejected by others (Ding et al., 2020; Zimmer-Gembeck et al., 2022), and felt self-esteem threats. The moderating effect of rejection sensitivity explained why some individuals were more likely to perceive the threat of neglect or rejection in social activities, and experience higher psychological pain in this process. In addition, those results also were consistent with views of the Rejection Sensitivity Model (Downey and Feldman, 1996; Levy et al., 2001). The Rejection Sensitivity Model emphasizes that how rejection sensitivity affects emotion-cognitive responses to social situations (Zimmer-Gembeck et al., 2022). In the model high rejection sensitivity will trigger individual anxious expectation of ostracism, which will result in negatively emotional reactivity (sadness, anger, and worry) and maladaptive behaviors (social withdrawal and aggressive behavior) (Zimmer-Gembeck, 2015). High rejection sensitivity adversely affects the establishment and maintenance of individual interpersonal relationships (Gao et al., 2017). In the present study, results indicated that high rejection sensitivity aggravated the negative effects of ostracism on self-esteem. In addition, consistent with the main views of the Self-verification Theory, individuals’ self-esteem and self-concept develops out of many sociocultural experiences (Korman, 1970; Swann, 1983, 1997). If individuals frequently confront ostracism, they would develop low self-esteem and low sense of self-worth. Lower self-esteem in turn, encourages individuals to withdraw from social interactions. Lower self-esteem is typical of cognitive bias due to rejection sensitivity. Individuals with high rejection sensitivity perceive more frequent rejections, experience more negative self-evaluation, reduce positive emotions and self-pleasure, and threaten their self-esteem (Li et al., 2019). Results of the present study expand the application of the Rejection Sensitivity Model in social situations. Rejection sensitivity is a vulnerable factor to interpersonal stress. Individuals’ rejection sensitivity will gradually form a self-defense mechanism, resulting in negative emotions and maladaptive behaviors, and will be further generalized to a wider range of social situations (Meadhan et al., 2018).

### Practical implications

For prevention and clinical implications, the present study focused on one specific outcome of ostracism: psychological defensiveness. First, the findings of this study suggest that, as a risk factor for adolescents, ostracism undermines adolescents’ perceptions of social resources. As a result, adolescents are more likely to respond defensively by withdrawal from social interaction following ostracism. They often deploy defensive strategies such as avoiding relationship with others (Bourgeois and Leary, 2001; Buckley et al., 2004). This suggests that intervention with the peer relationship is an important step to develop adolescents’ mental adaption. It is important to encourage adolescents to seek intimacy and create a vicious cycle of connecting with others. Second, Garfinkel (1956) described the Degradation ceremonies as formal rituals that remove a person from a valued role within a community. It is a common clinical observation of individuals who enact degradations on themselves. For example, ostracized individuals may rationalize the group that excluded them is one they would refuse to join. They could employ attempts of self-denouncement, and even draw others’ attention to their degradation for defensive purposes. It suggests that the defensive strategies may counter the negative effects of ostracism. Third, rejection sensitivity is a vulnerable factor to interpersonal stress. Individuals’ rejection sensitivity will gradually form a self-defense mechanism, resulting in negative emotions and maladaptive behaviors. Implications of the present study suggest that decreasing levels of rejection sensitivity may help individuals cope with ostracism. It suggests that teachers and parents should pay attention to the rejection sensitivity of adolescents, encourage adolescents to view negative events in life from a positive perspective. In addition, the present study found that the self-esteem partially mediated the association between ostracism and social withdrawal from the perspective of the Self-verification Theory. It suggests that in the process of dealing with the negative effects of ostracism, teachers and parents should pay attention to how to improve the level of self-esteem of adolescents. Educators can provide mentoring and training to adolescents to improve their self-esteem.

### The limitations and future research directions

The present study has several limitations. First, the study relies on self-reporting, and participants may choose more ethical options due to the social approval effect. Future studies can use multiple data sources (peer-reporting) and multiple measures (e.g., social measures, and experimental methods) to better measure adolescents’ perception of being ostracized. Second, as a cross-sectional study design study, it is impossible to clarify causal relationship between the research variables. However, the proposed moderated mediation model was based on a large number of theoretical and empirical evidence. In the future, longitudinal or experimental studies can be conducted to further examine the relationship between the variables in this study. Third, limited by the research conditions, the sample of this study is students from three high schools in Hubei and Shandong provinces, which may affect the generalization of the results. Future studies may examine the main findings of this study by selecting adolescents from different regions and school levels.

## Data availability statement

The raw data supporting the conclusions of this article will be made available by the authors, without undue reservation.

## Ethics statement

The studies involving humans were approved by the ethical institutional research committee of Hubei University of Art and Science. The studies were conducted in accordance with the local legislation and institutional requirements. Written informed consent for participation in this study was provided by the participants’ legal guardians/next of kin.

## Author contributions

YL: Conceptualization, Data curation, Formal analysis, Funding acquisition, Investigation, Methodology, Project administration, Resources, Software, Supervision, Validation, Visualization, Writing – original draft, Writing – review & editing. ML: Investigation, Methodology, Writing – review & editing. CL: Investigation, Methodology, Writing – review & editing. CZ: Investigation, Methodology, Writing – review & editing. ZY: Investigation, Methodology, Writing – review & editing.
